# Adapt or not: A comparison of rural migrant adaptation in two cities in China

**DOI:** 10.1371/journal.pone.0298238

**Published:** 2024-03-18

**Authors:** Jianxi Feng, Shuangshuang Tang

**Affiliations:** 1 Department of Urban Planning and Design, School of Architecture and Urban Planning, Nanjing University, Nanjing, China; 2 School of Geography, Jiangsu Center for Collaborative Innovation in Geographical Information Resource Development and Application, Nanjing Normal University, Nanjing, China; Nanjing Audit University, CHINA

## Abstract

Leading with the principle of ‘people-oriented urbanization,’ the adaptation of rural migrants in urban China has attracted increasing concerns from policy-makers and scholars. Today, China has proceeded to a new stage of urbanization. Many rural migrants prefer moving to cities near their home villages rather than to large cities, reflecting the changes in migration patterns and expectations of rural migrants. Although migrant adaptation has been repeatedly investigated in academia, researchers tend to address the topic in one host setting, while migrant adaptation in diverse urban settings has rarely been compared. This paper seeks to fill this research gap via a survey conducted in two cities with different urban settings in Jiangsu. The rural migrant adaptation experiences in the two cities are systematically compared. Our statistical results show that economic structure and living costs, on the one hand, and local regulations and socio-cultural environments, on the other hand, determine rural migrant adaptation experiences in different urban settings. Despite abundant employment opportunities in more-developed cities, the high living costs, working pressure, and strict institutional schemes significantly hamper rural migrant adaptation. In less-developed cities, limited employment opportunities and conservative socio-cultural environments hinder rural migrants from adapting in host societies. Our findings suggest that the governments of different cities need to tailor strategies to assist rural migrants in adapting in urban communities.

## 1. Introduction

Various studies have explored migrant adaptation in host societies [1–4]. The topic’s interest has increased rapidly in China since her rural-to-urban migration has become the largest globally and historically. By the end of 2019, 291 million rural migrant workers lived in urban areas [5], accounting for 21 percent of China’s total population (1.4 billion). Many rural migrants expect to adapt in cities and become urban citizens [6,7]. Nevertheless, the apparent inequalities between rural migrants and urban locals in China, including rights and benefits tied to institutional schemes, human capital, and social resources, significantly deter rural migrants from successfully adapting in urban communities [8]. The gaps between rural migrant expectations and reality may cause social instability. For instance, since 2010, several rural migrant workers in the Foxconn Electronics Company have committed suicide, revealing a pervasive sense of frustration and hopelessness among rural migrants in the city [9]. Given the enormous size and huge instability of this group, the adaptation of rural migrants in urban China has attracted policy-makers’ and scholars’ increasing attention in various disciplines.

In the existing literature, research on migrants’ adaptation has been conducted repeatedly. Nevertheless, the related studies mainly explore the experiences of migrants’ adaptation in one host society. Comparative studies of migrant adaptation in different settings remain scarce. Except for a few analyses making such a comparison at the country level [10,11], one at the city level is still lacking. Concerning rural migrant adaptation in Chinese urban settings, researchers find that rural migrants have difficulty adapting in host cities in general. In addition to the discriminatory *hukou* system, the disadvantaged human capital and social resources of rural migrants are key factors that hinder them from adapting in the mainstream societies [8,12–14]. Indeed, cities’ socio-economic settings interact with personal factors to influence rural migrant adaptation in a complex manner. But, little attention has been paid to the roles played by urban settings in rural migrant adaptation. Moreover, previous studies on the adaptation of rural migrants in Chinese cities mainly focused on those who work and tend to settle down in megacities along the eastern coastal area, such as Shanghai, Guangzhou, and Nanjing. Those cities have been the leading destinations for rural migrants since the beginning of massive urbanization. Nonetheless, the rate of urbanization in China has slowed down in recent years. The urbanization processes and patterns have also changed, revealing a new urbanization stage [15]. In China’s megacities, the soaring living costs and deceased opportunities brought by economic transformation make them no longer the first choice for rural migrants. Many rural people prefer to find jobs closer to their origins, especially in cities near their hometowns [16]. The central government also encourages rural migrants to settle in various types of cities rather than heading to the megacities in eastern China. Yet, existing literature rarely compares rural migrant adaptation in different kinds of cities in China’s new context.

With the new stage of urbanization and the changing preferences of rural migrants, several questions come up: what is the nature of rural migrant adaptation in recent years? Can rural migrants better adapt in their new destinations than into megacities? Are the determinants of migrant adaptation the same among cities at different development levels? These questions are pertinent to policy-makers in implementing their urbanization policies. Currently, the government focuses on the ‘quality’ rather than the ‘speed’ of urbanization. For the term ‘quality,’ the government is concerned with the process of how rural migrants adapt and settle in destination cities. Existing research mainly focuses on rural migrant adaptation in more-developed cities in eastern China during the first urbanization stage. Hence, a study comparing the rural migrant adaptation process and its determinants between different cities (for example, more-developed large cities in the eastern region vs. less-developed cities near rural hometowns) is necessary. The associated findings can help understand rural migrant adaptation in different urban settings under changing environments.

Based on a recent survey conducted in Jiangsu in China, this paper explores rural migrant adaptation in two types of cities in China’s current urbanization stage. It contributes to the existing literature on migrant adaptation by comparing the adaptation of rural migrants in diverse urban settings and investigating their adaptation determinants under China’s new settings. This paper is organized as follows: the literature on migrant adaptation and rural migrants in China is summarized in the following section. In section three, the study area and the data adopted in the paper are described. Section four compares the determinants of rural migrant adaptation experiences in Suzhou and Xuzhou based on regression models. Section five presents the conclusions and the implications for policy-making.

## 2. Literature review

### 2.1 Adaptation theories and empirical studies

The term adaptation is widely employed to describe the experiences of migrants in the host societies [1,8,17,18], referring to the changes that take place in individuals or groups as they attempt to come into harmony with new environments [19]. Previous research indicates that the experiences of adaptation are influenced by the socio-economic status of the migrants or groups and the host societies’ settings [20]. Both intrinsic and external factors affect migrant adaptation in new environments. Personal characteristics, such as income, educational attainment, language ability, job skills, and length of residence in the host societies, are commonly taken as the determinants of migrant adaptation in the host societies [3,20–23]. Concerning contextual factors, community characteristics are often mentioned. For instance, Zhou [2] found that community can moderate original cultural norms and create buffer zones to ease the tension between individuals and families in the host societies. Community-based support systems and positive cultural orientations rooted in the communities can prevent the younger generation from adapting to the host societies’ underprivileged segments [24]. In the Baltimore ghetto, strong social networks in the communities discouraged migrants and ethnic groups from accessing the opportunities outside, causing social isolation. Migrants or ethnic groups need to move away from ethnic enclaves to more diversified areas to better adapt in mainstream societies [24].

In contrast, very few studies analyze and compare the influences of the host societies’ diverse contexts on migrant adaptation. Among the existing studies, most focus on the contextual differences at the country level rather than the city level. For instance, Kilic and Menjívar [25] explored the fluid adaptation of second-generation Turkish immigrants in Berlin and New York. They found that the two countries have different immigration and citizenship laws, along with the related racialization practices and discriminatory treatment, all of which shape the experiences, practices, and senses of belonging differently for Turkish immigrants in different places. In New York, Turks have more flexibility to define themselves, while in Berlin, Turks are seen as outsiders. Leung [10] observed that Chinese migrant adolescents have a higher level of social support in Canada than in Australia, mostly due to large Chinese communities in Canada. In the communities, the importance of education is heavily emphasized, which increases parent-child tension and hurts Chinese migrant adolescents’ psychological adaptation accordingly.

In sum, based on theoretical and empirical research, it is found that the socio-economic status of migrants interacts with the settings of the host societies, influencing migrant adaptation. Nevertheless, most previous research on migrant adaptation has drawn upon empirical experiences in one host society. Despite a few studies comparing migrant adaptation in two host countries, migrant adaptation in different urban settings at the city level has been largely ignored.

### 2.2 Rural migrants and their adaptation in urban China

In China, rural migrants are often marginalized in cities, facing institutional, economic, social, and cultural barriers [8]. In prior studies, the *hukou* system is mostly regarded as the primary factor for the undesirable conditions of rural migrants in urban societies [8,26–28]. Due to institutional schemes, rural migrants could not benefit much from public welfare (e.g., social housing, public education for children) and state-provided opportunities [15,29,30]. For instance, restricted by the institutions, rural migrants have little chance to access low-cost housing, and a considerable number of them have to live in crowded dormitories provided by their employers or in shabby rental units that are isolated from urban residents in cities [31,32]. Although in Chongqing and Xiamen, migrants are allowed to apply for low-income housing [33], it is far from a nationwide practice.

Also, rural migrants tend to engage in the low-end manual jobs that urban residents dislike [34]. Such occupational segregation and wage differentiation are attributable to inadequate human capital (e.g., educational attainments and professional skills), the limited range of jobs migrants are allowed to take in host cities [29], and insufficient public channels for job-seeking information for the rural migrants. Instead of public sources, rural migrants’ job search relies heavily on friends and relatives [35]. The institutional barriers against rural migrants, the working and living isolation, and the social discrimination somewhat hinder the formation of effective social networks between rural migrants and urban residents in cities [36]. Non-native ties still account for most migrant networks in the receiving societies [12]. Consequently, rural migrants generally do not see themselves as urban residents, though they live and work in cities for a long time [8]. Their lack of sense of belonging dampens their adaptation in urban communities [37].

Many empirical studies explore the determinants of rural migrant adaptation in Chinese cities. The institutional barrier (the *hukou* system) is an important factor influencing migrant integration into urban societies [8]. In addition to discriminatory institutions, personal socio-economic factors also play important roles in rural migrant adaptation in urban areas. Wang and Fan [8] found that human capital is an essential indicator of migrants’ economic and identity adaptation, while dialect fluency and financial resources facilitate migrants’ social and cultural adaptation. Li et al. [14] illustrated that younger, more educated, and single rural migrant workers tend to adapt in Shenzhen’s urban societies. Higher-income and longer residence in cities facilitate their adaptation into the host cities. For social networks, social capital can help migrants adapt in urban societies [36]. Specifically, migrant-local ties are essential for rural migrant adaptation [13]. Nevertheless, Yue et al.[12] indicated that non-kin ties between migrants and local urban residents play a significant role in migrant adaptation, while non-resident ties still account for most migrant networks in China. At the community level, the residential and occupational types of migrant workers are associated with their social adaptation in cities [13]. In Wuhan, the type of residence (rental and self-owned housing) and the distance between living and working places positively influence the adaptation of rural migrant workers [38].

The above studies have shown that rural migrants in China often faced challenges in adapting in urban societies. This issue has attracted the increasing attention of scholars. However, the body of research on rural migrant adaptation is still scanty. Though some studies have concerned rural migrant adaptation in one host city, where the socio-economic status of rural migrants interacts with the settings of the host society, there is little research to compare rural migrant adaptation between different host urban societies. Likewise, since China has proceeded to a new stage of urbanization, there is a need to explore rural migrant adaptation determinants in the new setting. This paper aims to examine and compare the rural migrant adaptation in two types of cities in China’s new environments, in order to somewhat fill the research gap.

## 3. Methodology and data

### 3.1 Research area and survey

Our empirical analysis employs data from a survey in Jiangsu conducted between July and December in 2015, as part of a national grant project. The survey was designed to investigate the socio-economic and subjective features of rural migrants in Jiangsu’s urban areas.

Jiangsu locates in the most developed region in China, which is also a popular destination for rural migrants. Despite being the most affluent province in China, there is uneven development within the province. Jiangsu is divided into two sub-regions by the Yangtze River–the more developed south and the less developed north. Profound socio-economic disparities between the south and the north have existed over an extended period [39]. In this paper, two cities in Jiangsu, namely Suzhou (located in South Jiangsu) and Xuzhou (located in North Jiangsu), are selected as the research areas to compare rural migrant adaptation in different urban settings.

Suzhou and Xuzhou were selected as the study areas because they are at different development stages and have distinctive socio-economic features (see Table 1). Suzhou, located near the border between South Jiangsu and Shanghai, is the most affluent city in Jiangsu. In recent decades, foreign direct investment (FDI) and exports positively drive Suzhou’s development and influence the city’s industrial structure. In Suzhou, employments in primary industry account for only a small share of the manufacturing sector’s employment opportunities. The highly-urbanized and wealthy Suzhou strongly attracts rural migrants from inside and outside Jiangsu. In 2015, migrants accounted for ~40 percent of the total inhabitants (10.62 million) in Suzhou [40]. Despite abundant job opportunities and high-level earnings in the city, the working pressure, living costs, and institutional restrictions are much higher and stricter than those in the less-developed regions, which somewhat deter rural migrants from adapting in Suzhou.

**Table 1:** Socio-economic features of Suzhou and Xuzhou (2015).

	Rate of urbanization (%)	GDP per capita (RMB)	Employment structure (primary: secondary: tertiary)	Per capita disposable income of permanent urban residents (RMB)
Suzhou	74.9	136702	3.4: 60.0: 36.6	50390
Xuzhou	61.0	61511	31.7: 32.3: 36.0	26219

Source: Jiangsu Statistical Yearbook (2016).

Xuzhou is the largest city in North Jiangsu and is a traditional industrial base and an immense transportation hub, with dozens of railways and highways. It is also the primate city of Huaihai Economy Zone which is an underdeveloped area in China. The area is composed of 20 prefecture-level cities of four provinces: Xuzhou, Suqian and Lianyungang from the northern part of Jiangsu, Jining, Heze, Linyi, Zaozhuang, Rizhao, Taian and Laiwu form the southern part of Shandong, Huaibei, Suzhou, Fuyang, Bengbu, Bozhou from the northern part of Anhui, and Shangqiu, Zhoukou and Kaifeng from the eastern part of Henan. Multiple indicators of Huaihai Economic Zone such as residents’ income, urbanization rate and per capita GDP, are only 80% of the national average [40]. It is the underdeveloped area in the eastern and central part of China. Xuzhou is a popular destination for rural migrants of the surrounding area during the new stage of urbanization. Compared to Suzhou, Xuzhou is a less developed cities. Moreover, for the migrants from surrounding area, the cultural gap between their originations and host city is quite small. Some of them even have closes connections with local people. These factors will make the social adaptation process in Xuzhou is quite different from that in Suzhou. Hence, the two cities can somewhat represent two distinct types of cities–a more-developed large city in the developed regions and less-developed large cities near home villages of rural migrants.

The development stage and economic structure of the two cities-Xuzhou and Suzhou-are quite different. Compared to Suzhou, Xuzhou is less urbanized and holds a sizeable agricultural employment share. The GDP per capita (61,511 RMB) and the per capita disposable income of the permanent urban residents (26,219 RMB) in Xuzhou were approximately half of those in Suzhou in 2015 (136,702 RMB and 50,390 RMB, respectively) [40], revealing vast differences of the development stages between the two cities. In terms of economic structre, the GDP of manufacturing and construction sectors in Xuzhou is 234 billion RMB while the figure for Suzhou is 705 billion RMB, more than three times of that of Xuzhou. The highly developed secondary industry of Suzhou will provide more employment opportunities than that of Xuzhou for male migrants, given that they are mainly engaged in those sectors. Nevertheless, because of its size, location, and relatively low living costs, Xuzhou still attracts many rural migrants, especially those from nearby rural areas. Also, it attracts some return migrants from developed large cities in the eastern coastal regions.

The empirical analysis uses the data from a 2015 questionnaire survey of rural migrants in Suzhou and Xuzhou of Jiangsu. A stratified sampling method was adopted for the survey. First, four districts (Gusu, Huqiu, Wuzhong, and Wujiang) in Suzhou and four districts (Yunlong, Gulou, Quanshan, and Tongshan) in Xuzhou were chosen. Second, the questionnaires were distributed in the chosen districts. The Sixth National Census was employed to construct the sampling frame, focusing on the major sectors that hire rural migrants. According to the census, rural migrants were mainly engaged in the manufacturing, construction, and service sectors. In the survey, two types of sampling methods were used, one for the manufacturing and construction sector, and one for the service sector. For manufacturing and construction, we selected 16 industrial areas and six construction sites. For the other sectors, we selected 20 urban neighborhoods based on the population density and the distribution of rural migrants in that district. For each selected site, no more than 25 rural migrants were randomly surveyed. Finally, the survey’s total number of valid responses was 1,042, with 624 responses in Suzhou and 418 responses in Xuzhou.

### 3.2 Research methodology

This paper investigates the influences of urban settings on rural migrant adaptation by comparing the determinants of rural migrant adaptation in two cities with distinctive socio-economic features. Urban adaptation can be described with the following function:

Y=f(D,H,E,S,C,μ)

Here *Y* = urban adaptation, *D* = *Demographics*, *H* = *Human Capital*, *E* = *Economic Condition*, *S* = *Social Capital*, *C = Culture Maintenance*, *μ* = *unobserved factors*. Our research examines the impact of demographics, human capital, economic condition, social capital, and cultural maintenance on urban adaptation towards Suzhou and Xuzhou. To do so, we have pooled both sets of data in one set. Thus, the 624 observations from Suzhou were added to the 418 observations from Xuzhou, pro- ducing a pooled sample of 1024 observations. Dummy variables are used to test both independent and first-order interaction effects, based on the following equation:

Y=f(D,H,E,S,C,μ,DR,HR,ER,SR,CR,R)

with R functioning as a city dummy (0 for Xuzhou observations and 1 for Suzhou observations) while we test the difference between the intercepts. DR, HR, ER, SR and CR are interaction variables of the original independent variables multiplied with the city dummy variable R, testing the difference between the regression coefficients in the two years for each variable.

In this paper, the adaptation experiences of the rural migrants are the focus, capturing the true feelings of rural migrants in the host societies. Hence, the adaptation experiences of rural migrants were selected as the dependent variable, which comes from the following question in the questionnaire ‘Do you adapt well in the current city?’ The experiences of rural migrants were measured based on the ‘Satisfaction with Life Scale’ (SWLS) compiled by Diener et al [41]. Respondents rated the statement on a five-point ordinal scale from ‘strongly disagree’ (1) to ‘strongly agree’ (5).

Migrants and host urban backgrounds interact during adaptation process [2,42]. Both the socio-economic status of migrants and the host societies’ backgrounds influence migrant adaptation [20]. Migrant adaptation can be identified by multidimensional factors. To compare the determinants of migrant adaptation in these two cities with distinctive characteristics, factors in five dimensions, namely demographics, human capital, economic conditions, social capital, and cultural maintenance, which are highly associated with migrant adaptation, are included in the analysis framework. These selected indicators can signify not only personal characteristics but also contextual differences between the two cities. Therefore, they can compare the rural migrant adaptation experiences in different urban settings.

Specifically, demographic factors include age, gender, and marital status of rural migrants. Among those factors, age measures the cohort differences between the younger (born-after-1980) and the older generations. The cohort characteristics between these two groups of rural migrants are significantly different [43]. The human capital dimension of rural migrants contains two variables–educational attainment and residence duration. These variables are closely related to adaptation in the host societies through upward social mobility [2]. Economic status includes income and occupation. They are associated with the social status of migrants [44] and are linked with the destinations’ macro conditions, such as economic development and industrial structure [45]. The variable ‘owning an apartment in cities’ can somewhat represent the financial capability of rural migrants and their self-identity in the host societies [8,46]. It reveals the difficulty of becoming homeowners subject to the living costs in the city. It also indicates a vital stepping stone for rural migrants to become citizens in cities, associated with their self-identities [46]. Social capital is measured by rural migrant social contacts with local residents and family members at urban destinations. Social capital is an important indicator that influences migrant adaptation in the host societies [36]. Commonly, receptive environments established by the locals and the presence of family can facilitate the adaptation of migrants [2,18,47]. Specifically, in the paper social capital includes contract frequency with local residents, parents or siblings in destination, Spouses or children in destination. Finally, culture maintenance is linked with the adaptation of migrants. Normally, great cultural gaps may lead to poor adaptation for migrants [1]. In our analysis framework, the factor measures the cultural gaps between the origins and the destinations of rural migrants based on the divisions of cultural areas in China, indicating the cultural features of rural migrants and macro cultural backgrounds in the two destination cities.

## 4. Rural migrant adaptation in urban China

### 4.1 Descriptive analyses

Table 2 presents the characteristics of the sampled rural migrants in Suzhou and Xuzhou. We adopted a Chi-square test to compare and contrast the individual features and urban settings between Suzhou and Xuzhou. The self-evaluations of adaptation are based on a five-point scale ranging from 1 (strongly disagree) to 5 (strongly agree).

**Table 2:** Profile of rural migrants (%).

		Total	Suzhou	Xuzhou	Chi-square	*Sig*.
***Urban Adaptation***	Very low	1.8	2.6	0.6	83.286	0.000
	Low	8.3	11.4	4.2		
	Medium	25.8	33.0	16.0		
	High	49.9	46.2	54.9		
	Very high	14.2	6.8	24.3		
***Demographics***						
**Age**	< = 35 years	59.1	67.6	50.4	18.212	0.000
	>35 years	40.9	32.4	49.6		
**Gender**	Male	61.1	67.6	52.2	19.529	0.000
	Female	38.9	32.4	47.8		
**Marital status**	Married	57.4	56.7	58.5	0.289	0.591
	Single	42.6	43.3	41.5		
***Human Capital***						
**Education**	Primary school and below	12.5	13.8	10.7	25.950	0.000
	Middle school	65.2	70.2	58.2		
	College and above	22.3	16.0	31.1		
**Residence duration**	<1 year	19.7	19.9	19.6	0.401	0.818
	1–5 years	48.8	49.5	47.8		
	>5 years	31.5	30.6	32.6		
***Economic Condition***						
**Monthly income**	<4,000 RMB	32.5	59.3	21.1	34.586	0.000
	>4,000 RMB	67.5	40.7	78.9		
**Occupation**	Workers	48.7	50.3	46.6	2.987	0.394
	Service personal	28.8	28.0	29.7		
	Clerical personal	13.1	11.6	15.1		
	Self-employed	9.4	10.1	8.6		
**Owning an apartment in Suzhou/Xuzhou**	Yes	22.6	11.8	37.4	72.634	0.000
	No	77.4	88.2	62.6		
***Social Capital***						
**Contact frequency with local residents**	Low	19.2	22.5	14.8	141.659	0.000
	Medium	50.7	63.9	32.7		
	High	30.1	13.6	52.5		
**Parents or siblings in destination**	Have	30.2	26.3	35.6	8.153	0.004
	Have not	69.8	73.7	64.4		
**Spouses or children in destination**	Have	42.0	39.8	45.1	2.295	0.130
	Have not	58.0	60.2	54.9		
***Culture Maintenance***						
**Cultural gap**	High	65.7	94.1	24.3	444.354	0.000
	Medium	12.1	1.4	27.9		
	Low	22.2	4.5	47.8		
***Number***		1,042	624	418		

Generally, the rural migrant adaptation experiences in Suzhou and Xuzhou are significantly different. Rural migrants in Suzhou reported a much lower level of adaptation experiences than do those in Xuzhou, as 79.2 percent of rural migrants in Xuzhou pointed out that they adapted well in the city. In contrast, approximately half of the rural migrants in Suzhou (53.0 percent) hold the same opinion. Further, only 4.8 percent of the Xuzhou respondents mentioned that they had poor adaptation experiences, whereas the percentage was 14.0 for rural migrants in Suzhou. Compared with rural migrants in Suzhou, those in Xuzhou seem to better adapt in their host societies. The results reveal the differentiated adaptation process of rural migrants between cities at different development stages. Considering the locations of cities and the origins of rural migrant respondents, it is observed that rural migrants can better adapt in less-developed large cities near their rural hometowns than in more-developed large cities in eastern China.

Compared with those in Xuzhou, the sampled rural migrants in Suzhou were mainly younger men with higher educational attainments, indicating the two cities’ labor structure disparities. A larger share of respondents in Suzhou earned a salary of >4,000 RMB per month. At the same time, the number of homeowners in Xuzhou was much higher than that in Suzhou. The significant disparities of income and urban housing ownership of the rural migrants may reflect the different development stages between the two cities. In Suzhou, abundant employment opportunities (especially in the manufacturing sector) and high-level wages attract the in-migration of rural migrants. Nevertheless, the living costs are soaring in Suzhou, probably hindering rural migrants from residing there permanently. Concerning social resources, respondents in Xuzhou interacted with the local residents more frequently than those in Suzhou, and a larger share of them had family members in the host cities. In terms of cultural maintenance, most rural migrants had similar cultural features to the Xuzhou urban locals. In contrast, more than half of the rural migrants encountered huge cultural gaps in Suzhou. The two indicators may reveal that the rural migrants in Xuzhou live in more socially and culturally familiar environments than those in Suzhou. The environments likely help them adapt in urban communities.

### 4.2 Regression analyses

The previous sections indicate that the socio-economic features of Suzhou and Xuzhou and the adaptation experiences of rural migrants in the two cities are significantly different. In this section, we adopted regression models to investigate the influences of different urban settings by comparing the determinants of the rural migrant adaptation experiences and analyzing the differences between them. The results are reported in Table 3. It shows that the dummy variable (Xuzhou) is positively significant, suggesting an increased likelihood of rural migrant adaptation in Xuzhou as compared to Suzhou.

**Table 3:** Regressions on adaptation experiences of rural migrants.

Variable	Total	Coefficients of Suzhou	Changes in Coefficients	Coefficients of Xuzhou
**Xuzhou (dummy)**	--	--	0.512**(2.395)	--
***Demographics***				
**Age**				
>35 years	-0.063(-1.595)	-0.039(-0.689)	-0.036(-0.524)	-0.093(-1.405)
< = 35 years (= ref.)				
**Gender**				
Male	-0.007(-0.204)	0.031(0.637)	-0.080(-1.330)	-0.072*(-1.275)
Female (= ref.)				
**Marital status**				
Married	-0.063(-1.387)	-0.058(-0.987)	-0.016(-0.198)	-0.086(-1.065)
Single (= ref.)				
***Human Capital***				
**Education**				
Middle school	-0.048(-1.019)	-0.067(-1.079)	0.063(0.703)	0.003(0.039)
College and above	0.004(0.078)	-0.019(-0.262)	0.045(0.518)	0.045*(0.483)
Primary school and below (= ref.)				
**Residence duration**				
1–5 years	0.091*(2.175)	0.105*(1.919)	-0.003(-0.040)	0.114(1.565)
>5 years	0.137**(2.991)	0.118*(1.931)	0.042(0.590)	0.197**(2.519)
<1 year (= ref.)				
***Economic Condition***				
**Monthly income**				
>4,000 RMB	-0.040(-1.177)	-0.020(-0.471)	-0.009(-0.201)	-0.034(-0.593)
<4,000 RMB (= ref.)				
**Occupation**				
Workers	0.090*(1.769)	0.151*(2.081)	-0.123*(-1.469)	-0.004(-0.044)
Service personal	0.022(0.532)	0.039(0.688)	-0.018(-0.334)	0.012(0.181)
Clerical personal	0.048(0.997)	0.066(1.007)	-0.037(-0.521)	0.017(0.219)
Self-employed (= ref.)				
**Owning an apartment in Suzhou/Xuzhou**				
Yes	0.147***(4.216)	0.252***(4.049)	-0.180**(-2.695)	0.059(1.059)
No (= ref.)				
***Social Capital***				
**Contact frequency with local residents**				
High	0.245***(5.882)	0.305***(6.017)	-0.163**(-2.578)	0.071*(0.921)
Medium	0.354***(7.920)	0.428***(6.346)	-0.217**(-2.490)	0.230**(2.873)
Low (= ref.)				
**Parents or siblings in destination**				
Have	0.060*(1.836)	0.059(1.316)	-0.003(-0.061)	0.065(1.189)
Have not (= ref.)				
**Spouses or children in destination**				
Have	0.164***(3.734)	0.170**(2.903)	0.005(0.064)	0.198**(2.686)
Have not (= ref.)				
***Culture Maintenance***				
**Cultural gap**				
High	-0.207***(-5.070)	-0.074*(-0.636)	-0.048(-0.577)	-0.151**(-2.569)
Medium	-0.033(-0.908)	0.067(0.308)	-0.090(-0.412)	-0.037(-0.644)
Low (= ref.)				
**Adjusted R** ^**2**^	0.251	0.255	--	0.181
**Number**	1,042	624	--	418

***p<0.001

**p<0.05

*p<0.01.

#### 4.2.1 Determinants of rural migrant adaptation experiences

In this section, the determinants of rural migrant adaptation experiences in the most recent urbanization stage in China are investigated. As shown in Table 3, the length of residence in the host city is positively associated with the adaptation experiences of rural migrants. Longer residence may increase the exposure of migrants to local urban cultures and the accumulation of social capital and economic resources in cities, facilitating adaptation [14]. It also strengthens rural migrants’ attachment to the cities, which is related to social stability, familiarity, and security [48].

In terms of economic well-being, self-employment is negatively associated with the adaptation experiences of rural migrants. In cities, many rural migrants are self-employed, mainly because of their limited human capital. Typically, those self-employed rural migrants have no urban insurances, and they tend to engage in informal sectors bounded by local regulations and laws [49]. Also, rural migrants are more likely to encounter discriminatory policies when applying for business licenses and dealing with commercial and tax issues [50]. In recent years, rural migrants are more willing to settle down in cities rather than floating between urban destinations and rural origins. Nevertheless, the conditions of self-employed rural migrants are still disadvantaged ***and*** without considerable amelioration in recent years. Consequently, the persistent unstable situation reduces the adaptation experiences of self-employed rural migrants in the host cities.

Recently, migrants are allowed to apply for social housing in some Chinese cities. Nevertheless, the policy has not been carried out nationwide [33]. Under current institutional systems, rural migrants are still largely excluded from social housing provision in the host cities [8]. In addition, rural migrants are often unable to apply for financial institutions’ credit support because of their low incomes and unstable conditions [51]. Subject to the control of housing purchases in recent years, rural migrants face stricter requirements (e.g., have been paying tax or persistent urban insurance over some years) in buying urban housing than the locals face. In cities, rural migrant homeowners are often elite groups with relatively abundant financial and social resources [52,53]. Besides, having permanent homes in cities implies a strong desire to settle down and become urban citizens [46]. Hence, from both economic and psychological perspectives, it is reasonable that owning an apartment in the host cities is positively related to the adaptation experiences of rural migrants.

In the new urbanization stage, family members’ social networks are still a significant predictor for rural migrant adaptation in host cities. There are several possible explanations. First, due to limited information channels at urban destinations, the kinship ties of rural migrants, including family members and relatives, are crucial resources in job-seeking and accommodations in new environments [24,54]. Second, social supports from households play an essential role in stress relief and emotional support in the adaptation in urban communities [24]. Nevertheless, rural migrants need to broaden their social ties outside their households (and communities) if they want to adapt in the host societies. In the model, interactions with the local residents significantly affect the adaptation experiences of rural migrants. It shows that the more frequently rural migrants come in contact with the locals, the more likely they are to accept the host cultures and adapt in the host societies. This echoes with previous research findings [55,56]. The factor ‘cultural gap’ hinders the adaptation of rural migrants. The cultural gaps represent the cultural differences or dissimilarities between the place of origin and the destination. Typically, a great cultural gap and a negative attitude towards the host cultures may induce cultural conflicts, leading to poor adaptation [1]. In other words, the familiar cultures of the receiving societies can help rural migrants adapt to urban lives socially and psychologically. In the process of acculturative transition, rural migrants may build their social identities and a sense of belonging toward the city [57].

Moreover, it has to be noted that the coefficients of social and cultural factors in the model are large. It probably indicates that, in the new urbanization stage, when ‘quality’ is emphasized to a greater extent than before, rural migrants expect a more socially and culturally receptive environment. Unlike being isolated in migrant communities, rural migrants can genuinely develop an attachment to the receiving societies by interaction with the locals and acculturation, further increasing their intentions of settling down in the host cities [58].

#### 4.2.2 Disparities of the determinants of rural migrant adaptation experiences between Suzhou and Xuzhou

Despite some similarities in the model, the results show the substantial disparities of the determinants of rural migrant adaptation experiences in the two cities. Compared with women, men are more likely to have worse adaptation experiences in Xuzhou, while gender has no significant effect in Suzhou. The change in coefficient for the variable is the opposite of Suzhou, suggesting a decrease in men’s adaptation experiences in Xuzhou. The results are probably attributable to the different development stages and employment structures of the two cities. Typically, the labor markets of rural migrants are highly segregated by gender. Male rural migrants tend to engage in the manufacturing and construction sectors. Suzhou could provide abundant employment opportunities in secondary industry for rural migrants because it is a large manufacturing base and a rapidly-expanding city. In contrast, the jobs in Xuzhou are relatively limited and low-paid. Hence, men are less likely to find suitable jobs in Xuzhou than in Suzhou. Given that the male is usually the breadwinner in the households, fewer formal job opportunities for the male in the host cities may adversely affect their adaptation [59].

The educational attainment of college and above has a significant positive effect on the assimilation experiences of rural migrants in Xuzhou, while the effect is insignificant in Suzhou. Being a developed large city, Suzhou is a popular destination for talents, while Xuzhou is less attractive for those high-ranking people. Consequently, in the environments with less fierce competition (such as Xuzhou), highly educated rural migrants are more likely to have higher social status and find better jobs, achieving better assimilation. In Suzhou, rural migrants typically have difficulty meeting their expectations, thereby dampening their adaptation experiences.

Workers are more likely to have better adaptation experiences than self-employed rural migrants in Suzhou. Nevertheless, the coefficient change indicates a negative impact on the rural migrant adaptation in Xuzhou. On the one hand, in Suzhou, self-employed rural migrants usually have to accumulate more financial resources to run their business because of the high operating costs in an affluent city. On the other hand, administrative management in Suzhou, including business licenses and tax issues, is stricter than in Xuzhou because those regulations are commonly associated with the city’s attractiveness in China. Normally, the more migrants a city attracts, the more stringent the control policies towards migrants will be [60]. As a consequence, self-employed rural migrants in Suzhou may encounter more difficulties in maintaining a stable condition. On the contrary, although rural migrant workers are often deprived of benefits and leisure time in global production facilities, their employment status seems more stable than self-employed rural migrants in Suzhou, thereby increasing their sense of adaptation. Compared with the rural migrants in Suzhou, those in Xuzhou are less affected by high-pressure production regimes and stringent urban regulations. The disparities of working conditions of the rural migrants engaging in different sectors between the two cities are not obvious. Hence, the effect of ‘occupation’ on the adaptation experience is weaker.

Concerning economic conditions, owning apartments in Suzhou has a significant and positive effect on the adaptation experience of rural migrants. Nevertheless, in Xuzhou, the effect is significantly diminished. As mentioned above, rural migrant homeowners in urban destinations usually have the most human capital and financial resources within the rural migrant groups. The disparity between homeowners and non-homeowners is even wider in affluent large cities since urban housing prices keep soaring. In Suzhou, urban housing costs much more than in Xuzhou. Specifically, in 2015, the average prices of commodity housing in Suzhou were ~RMB11,500 /m^2^, in contrast to ~RMB5,770/m^2^ in Xuzhou. Rural migrants in Suzhou have to pay twice as much to buy an urban apartment. Typically, in Suzhou, most rural migrants cannot afford the high housing price to become homeowners. Thus, owning an apartment in Suzhou, representing high socio-economic status, significantly strengthens the sense of belonging and urban identities of the rural migrants in the receiving societies [8]. In contrast, due to the reduced difficulty in becoming homeowners in Xuzhou, owning an apartment has no significant effect on the adaptation of rural migrants.

Contact with local residents is positively associated with the sense of adaptation in the two cities. It indicates that migrant-locals ties play an important role in rural migrant adaptation, echoing a previous study [12]. Nevertheless, such an effect is much stronger in Suzhou. As shown in Table 2, rural migrants’ contacts with the locals are much less frequent in Suzhou than in Xuzhou. That is probably because, in Suzhou, rural migrants are geographically confined to workplaces. They live in dormitories or migrant enclaves. Consequently, rural migrants in Suzhou tend to be segregated from the locals and have homogeneous social networks within the migrant communities. In this context, ties between migrants and locals seem more valuable for the rural migrants in Suzhou to become familiar with the host societies, thereby contributing more to the adaptation experiences. In more socially familiar environments (such as Xuzhou), perhaps only the establishment of close and instrumental ties with the locals can truly enhance the adaptation of rural migrants.

Concerning cultural maintenance, the huge cultural gaps have a strong negative impact on the adaptation experiences of rural migrants in Suzhou and Xuzhou. The change in coefficient indicates an increasingly negative effect on the sense of adaptation in Xuzhou. That is probably because migrant adaptation could be achieved more efficiently in multicultural settings where numerous migrants with diverse cultural backgrounds are living [1]. Despite limited contacts with the locals, a sense of ‘intercultural belonging’ can be cultivated through rural migrants’ daily experiences [61]. In a traditional society, where a large share of residents have similar cultural backgrounds (such as Xuzhou), rural migrants with huge cultural gaps are more likely to be marginalized, thereby weakening their sense of belonging.

## 5. Conclusion

With the increasing number of rural migrants in China, migrant adaptation has attracted growing attention from both scholars and policy-makers. The existing literature mainly focuses on the experience of migrant adaptation in one host society, while comparisons of migrant adaptation in different urban settings at the city level are scanty. Also, China has proceeded to a new stage of urbanization. Rural migrants may have different destination preferences and adaptation requirements in different urban settings. This study sought to fill this research gap by comparing the rural migrant adaptation experiences in different urban settings, based on a recent survey conducted in two types of cities in Jiangsu in China. Our findings contribute knowledge to the conditions and situations of migrant adaptation in different urban settings, which also deepens our understanding of rural migrant adaptation in Chinese cities.

In the paper, the factors of five dimensions are included to examine their influences on rural migrant adaptation experiences. Our findings reveal that human capital, economic condition, social networks, and culture maintenance are important determinants for rural migrant adaptation. Consistent with the findings of previous studies [8,12–14], factors such as long residence, homeownership, and the presence of family members in the host cities significantly facilitate rural migrant adaptation in the host cities. On the other hand, financial resources and kinship networks play key roles in improving rural migrants’ survival likelihood and upward social mobility, enhancing their adaptation in urban communities. Compared to those factors, ‘contacting local residents’ and ‘small cultural gap’ have more significant impacts on rural migrant adaptation in the host cities. That is probably because the expectations of rural migrants have been changing. Currently, a considerable number of rural migrants want to become urban citizens. Many of them treasure the quality of life on top of earning a living [16]. Hence, apart from economic adaptation, socio-cultural adaptation becomes crucial for rural migrants to develop their sense of belonging to the host cities. Through ‘mixing’ with the locals and acculturation, rural migrants familiarize themselves with the cities and build their urban identities, facilitating their adaptation.

More importantly, our empirical analysis indicates that the determinants of rural migrant adaptation experiences vary in different cities. Specifically, migrants’ demographics, human capital, economic condition, social capital and culture maintenance, interacting with urban settings like industrial structure, local regulations and socio-cultural environments, tend to influence migrants adapation in different ways. Our analyses indicate that due to the industrial structure, the soaring living costs, fierce competition, and strict regulations in Suzhou, female migrants, self-employed migrants, migrants with higher educational attainment, no apartment and weak migrant-locals ties are more likely to have worse adaptation experiences than thoese in Xuzhou. In the new stage of urbanization process, many rural migrants choose to leave more-developed large cities and move to those near their hometowns despite the abundant employment opportunities and relatively high wages in the former. In this circumstance, large cities close to rural migrants’ hometowns become decent choices. Nevertheless, our findings reveal that rural migrants still encounter difficulties in adaptating in those cities. Although the low living costs and loose institutional schemes in less-developed large cities (like Xuzhou) help rural migrants to settle down and achieve upward social mobility, the limited jobs and earnings there somewhat confine the opportunities of rural migrants to work and reside permanently. What is more, those cities tend to be acquaintance and homogenous societies, in which culture and social ties play crucial roles in people’s daily lives. Hence, newcomers may encounter huge barriers in familiarizing themselves with those cities if they do not have adequate instrumental social resources or refuse to embrace the local cultures. Therefore, we cannot merely state that rural migrants who are excluded from more-developed large cities can easily adapt to the societies in less-developed cities. Both opportunities and drawbacks for rural migrant adaptation exist in different urban settings.

Our findings suggest that policy-makers need to set targeted pathways to assist rural migrants in adapting in different urban societies. Despite a series of *hukou* reforms, governments in more-developed large cities tend to behave entrepreneurially, aiming at attracting high-level talents, while rural migrants are largely overlooked. For instance, in Beijing, many low-end migrants are driven out during shantytown reconstruction. In Shenzhen, many urban villages, which are shelters for rural migrants, are demolished without providing compensated accommodations. Indeed, the basic needs of rural migrants, who are contributors to their host cities, including social security and housing, should be catered by the governments. In addition, socially and culturally friendly environments are needed to offset the adverse effects of high living costs and working pressures on rural migrants. In less-developed cities, the provision of employment opportunities is a primary concern. If more decent and stable jobs can be offered, the low living costs and loose institutional schemes in less-developed cities will attract rural migrants, especially those from the surrounding rural areas. Because of the geographic proximity, similar cultural backgrounds and existing social capital can help those rural migrants from nearby get used to urban cultures and lifestyles, cultivating their sense of belonging to a greater degree. Overall, those policies would help improve the well-being of rural migrants in urban societies and accelerate the ‘people-oriented’ urbanization of China in the new era.

There are a few limitations of the study. First, the lagging data we used may not capture some new intentions of rural migrants and new trends of urbanization occurred in current China. In the future we will employ new data to detect the urban adaptation of rural migrants. Second, we only choose Suzhou and Xuzhou as the destination cities of rural migrants. Both of them are big cities from Jiangsu Province, which cannot represent all types of destinations of rural migrants, for instance, small cities in less developed area. To get full and perhaps deep understanding of social adaptation of rural migrants in host cities, more sophisticated research with well-designed data is needed in the future.

## Supporting information

S1 Dataset (SAV)
